# School playgrounds and physical activity policies as predictors of school and home time activity

**DOI:** 10.1186/1479-5868-8-38

**Published:** 2011-04-27

**Authors:** Rachael W Taylor, Victoria L Farmer, Sonya L Cameron, Kim Meredith-Jones, Sheila M Williams, Jim I Mann

**Affiliations:** 1 Edgar National Centre for Diabetes and Obesity Research, Department of Medical and Surgical Sciences, University of Otago, PO Box 913, Dunedin 9054, New Zealand; 2 Department of Human Nutrition, University of Otago, PO Box 56, Dunedin 9054, New Zealand; 3 Department of Preventive and Social Medicine, University of Otago, PO Box 913, Dunedin 9054, New Zealand

## Abstract

**Background:**

Previous work has suggested that the number of permanent play facilities in school playgrounds and school-based policies on physical activity can influence physical activity in children. However, few comparable studies have used objective measures of physical activity or have had little adjustment for multiple confounders.

**Methods:**

Physical activity was measured by accelerometry over 5 recess periods and 3 full school days in 441 children from 16 primary schools in Dunedin, New Zealand. The number of permanent play facilities (swing, fort, slide, obstacle course, climbing wall etc) in each school playground was counted on three occasions by three researchers following a standardized protocol. Information on school policies pertaining to physical activity and participation in organized sport was collected by questionnaire.

**Results:**

Measurement of school playgrounds proved to be reliable (ICC 0.89) and consistent over time. Boys were significantly more active than girls (P < 0.001), but little time overall was spent in moderate-vigorous physical activity (MVPA). Boys engaged in MVPA for 32 (SD 17) minutes each day of which 17 (10) took place at school compared with 23 (14) and 11 (7) minutes respectively in girls. Each additional 10-unit increase in play facilities was associated with 3.2% (95% CI 0.0-6.4%) more total activity and 8.3% (0.8-16.3%) more MVPA during recess. By contrast, school policy score was not associated with physical activity in children.

**Conclusion:**

The number of permanent play facilities in school playgrounds is associated with higher physical activity in children, whereas no relationship was observed for school policies relating to physical activity. Increasing the number of permanent play facilities may offer a cost-effective long-term approach to increasing activity levels in children.

## Background

Despite current controversy over the relative contribution of physical activity to weight management during growth [1,2], it is clear that physical activity has numerous other physical and mental health benefits [3]. Secular decreases in physical activity in children have increased the need for effective strategies which encourage and promote active lifestyles [4]. Schools provide an integral setting for the promotion of physical activity through many avenues including physical education classes, extra-curricular sports, recess, supporting community initiatives and promulgating appropriate policies [5-7].

Despite the acknowledged need for appropriate physical activity policies [8], difficulties in implementation are apparent [9,10]. Although studies have demonstrated that school activity policies do influence physical activity levels [11-13], few studies have utilized an objective method of physical activity assessment or physical fitness [14].

Because of intensive demands on school time, exploration of the efficacy of non-curricular approaches to increasing physical activity is also warranted [15]. Initiatives to increase physical activity during recess have focused on improving physical education classes [16,17], adding playground markings [18,19] or extra portable sports equipment [20], or providing structured play sessions from additional staff [21,22]. Little research has investigated the ability of more permanent playground changes to influence physical activity in the long-term [23]. Evaluation of a single playground demonstrated that children were more likely to be active in the more equipped areas [24], and school facilities have been associated with activity in young children [25]. We have previously shown that the number of permanent play facilities in school playgrounds is associated with physical activity measured using accelerometry [26]. However, this study only included a small sample of schools (n = 7) and playground counts were only ascertained by one researcher.

Therefore, the aims of this study were firstly to determine if the number of permanent play facilities in school playgrounds and the presence of school physical activity policies were independently associated with higher levels of physical activity in children once appropriately adjusted for confounders, and secondly, to assess the reliability of a playground measurement tool.

## Methods

All primary schools (Years 1 to 8 inclusive) in the greater Dunedin area with school rolls of more than 150 pupils were eligible to participate in the PLAY study (n = 21). The study was approved by the University of Otago Ethics Committee (09/052). Schools provided written informed consent to participate in the study, as did parents of eligible children. The children themselves gave verbal consent after consulting their parent/guardian. Each participating school was asked to nominate one Year Two and one Year Four class and all children in these classes were invited to participate in the individual level measurements. Children with physical disabilities that prevented normal play were excluded from participating. Information on school decile rating was obtained from the 2008 Ministry of Education database. A school's decile indicates the extent to which it draws its students from low socio-economic communities; decile 1 schools are the 10% of schools with the highest proportion of students from low socio-economic communities whereas decile 10 schools are the 10% of schools with the lowest proportion of these students http://www.minedu.govt.nz.

### Measurements in schools

The number of permanent play facilities in school playgrounds was measured using a protocol developed for this study and pre-tested in three schools not included in these analyses. Researchers (hereafter referred to as "raters") walked around the schools and recorded each individual item that could be used by children for physical activity according to predefined guidelines. Counts were obtained for separate items divided into 23 categories including objects such as swings, climbing frames, courts, goals, slides, nets, playground markings, swimming pools and so on. Guidelines were provided to indicate how to divide particular types of equipment into counts, given the variability in size of some equipment (climbing frames/walls, nets, fort platforms, bars, and bridges). Measurements of playground counts were obtained in each school on 3 separate occasions, separated by at least one week, by 3 researchers, resulting in 9 measures per school.

The extent of existing school policies pertaining to physical activity was assessed by a questionnaire modified for the New Zealand education environment from the Centers of Disease Control School Health Index http://www.cdc.gov. The questionnaire asked about the extent and quality of physical education classes (items PA.1, PA.2, PA.3, PA.4, PA.5, PA.6, PA.9 and PA.10 from Module 3 from the School Health Index) and recess (PA.1 from Module 1), the adequacy and availability of school facilities during school and after hours (PA.2 and PA.3 from Module 1), promotion of physical activity for all regardless of skill level (PA.7 and PA.11 from Module 3), policies around use of physical activity as a punishment or reward (PA.4 from Module 1), the reach of school activity into the community (PA.8 from Module 3), and safety issues (PA.12 and PA.13 from Module 3 and S3 from Module 1).

### Measurements in individual children

Birth date, sex, and ethnicity were obtained from school records. Duplicate measures of height (Seca 220, Wedderburn, Dunedin) and weight (Tanita HD-316) were obtained during class time on Monday mornings and body mass index (BMI, kg/m^2^) was calculated. US reference norms [27] were used to determine BMI z-score and to classify children as overweight (≥ 85^th ^but < 95^th ^BMI percentile) or obese (≥ 95^th ^percentile). Physical activity was assessed using ActiGraph GT3X accelerometers (ActiGraph, Pensacola, FL), set at 60-second epochs. Researchers fitted the accelerometers to the children on the Monday morning, placed at the waist in line with the right knee. Children were advised to wear the accelerometers during all waking hours (except for swimming and bathing) until they were removed by the researchers on the Friday afternoon of that week. Parents completed a daily accelerometer form which recorded 1) participation in organized sport (type and minutes), 2) how children had travelled to and from school (car, bus, walk, bike, other), 3) the time the child went to bed, went to sleep, and got up each morning, and 4) whether the accelerometer was removed for any reason and for how long.

### Data reduction

ActiGraph data were analyzed using MeterPlus (Santech Inc, La Jolla, CA). This program allows batch scoring of data, the exclusion of data when non-worn (defined as a bout of "0" counts for at least 20 minutes), and time filters as appropriate. A day was only considered valid if more than 8 hours of data were obtained [28]. Because of variation in sleeping habits in children, all data were analyzed from 8 am to 8 pm. The day was divided into school time (9 am-3 pm in New Zealand schools) and home time (8 am-9 am and 3 pm-8 pm) activity [26] and data were available for 3 full days per participant (Tuesday, Wednesday and Thursday). Physical activity during recess time only (a short morning break and a longer lunch break) was also calculated and this was available over 5 days of measurement (Monday to Friday inclusive). Participation in moderate-vigorous activity (MVPA) was defined according to the cutoffs of Puyau *et al *[29]. Daily weather data (rainfall, minimum and maximum temperatures, wind) was obtained from the Metservice website http://www.metservice.co.nz.

### Statistics

Because the sampling unit was school, a mixed model with random effects for school, rater and time was used to estimate the reliability of the playground equipment count. This takes into account any correlation among repeated ratings or ratings of the same equipment. The rest of the data were analyzed using the survey command from Stata 11.1 (College Station, TX: Stata Corporation 2010) with school as the primary sampling unit. This allows for the complex sample. As the accelerometry data were log transformed before analysis the results are presented as ratios for an increase of 10 units of play equipment. A ratio of 1.05, for example, represents a 5% increase in the outcome variable for 10 pieces of play equipment. No adjustment was made for multiple testing.

## Results

473 children consented to participate in the measurements (response rate 66%) with some variation in numbers between schools (Table 1). The number of playground facilities in the 16 schools ranged from 30 to 135, with an average of 89 (SD 22) and school policies on physical activity also varied between schools (81 (6)). As expected, larger schools tended to have more facilities although this was not significant (r = 0.476, P = 0.63). The intra-class correlation of the playground counts was 0.89, demonstrating that our measure of playground counts was both consistent and reproducible.

**Table 1:** Characteristics of the schools

School	Roll	School decile^1^	Response rate (%)	Number consented	Number measured	Playground counts	PA policy
1	253	10	80	35	35	88	75
2	166	2	60	17	14	86	78
3	230	8	70	35	32	135	69
4	158	10	72	30	29	30	80
5	190	3	64	30	25	73	88
6	287	6	67	26	26	112	88
7	324	9	73	35	33	89	80
8	367	10	56	25	22	113	78
9	160	10	89	40	36	95	77
10	204	4	69	32	31	96	88
11	295	9	78	38	35	91	88
12	240	10	67	29	28	96	86
13	212	8	53	23	23	73	84
14	151	5	57	26	23	76	82
15	250	9	65	32	31	77	84
16	293	4	45	21	18	90	73

^1^School decile indicates the extent to which a school draws its students from low socio-economic communities; decile 1 schools are the 10% of schools with the highest proportion of students from low socio-economic communities whereas decile 10 schools are the 10% of schools with the lowest proportion of these students.

Table 2 presents the characteristics of the 441 consented children who were present on the day of measurement. Height, weight and BMI were similar between boys and girls and approximately one in four children were classified as overweight or obese. Children were predominately Caucasian (91%), with 7% identifying as Maori and 2% as Pacific Islanders. Children who consented to participate but who were absent on the day of measurement (n = 32) did not differ from children who were measured in either age (P = 0.686), sex distribution (P = 0.205) or school decile rating (P = 0.158). One hundred and thirty boys (49%) and 109 girls (50%) participated in organized sport over the week of measurement for an average of 103 (SD 77) minutes. Children who participated in organized sport were not more active overall (counts/minute) than children who did not participate (P = 0.258), although they did engage in 3 additional minutes of MVPA each day (P = 0.029).

**Table 2:** Body size and physical activity of participants

	Boys	Girls	P
n	235	206	
Age (years)	8.0 (1.1)	8.1 (1.1)	0.610
Height (cm)	129.5 (8.4)	128.4 (8.0)	0.059
Height z-score	0.33 (0.93)	0.13 (0.90)	<0.001
Weight (kg)	29.3 (7.0)	28.6 (6.0)	0.234
Weight z-score	0.54 (0.86)	0.38 (0.83)	0.022
BMI (kg/m^2^)	17.2 (2.3)	17.2 (2.1)	0.911
BMI z-score	0.51 (0.81)	0.46 (0.75)	0.530
Overweight or obese n (%)*	60 (25.5)	48 (23.3)	0.614

All data are presented as mean (SD) except where stated.

P for difference between boys and girls.

*According to US reference norms [27].

Valid accelerometry data on every day of measurement were obtained from 380 children and from a further 47 children over two days. Children with valid accelerometry data (n = 380) did not differ from children with incomplete data (n = 61) for age (P = 0.344), BMI (P = 0.753) or sex distribution (P = 0.391). Data collection during recess was also high; 404 children (92%) wore the accelerometers for at least 80% of the recess time. Table 3 shows the average counts per minute and daily minutes of MVPA during the total day, as well as the defined periods of recess, school day (9 am-3 pm), and home time (8-9 am and 3-8 pm). Regardless of the measure (counts or MVPA) or time frame (recess, school, home) boys were more active than girls, although the differences were often small. Boys spent 9 more minutes each day engaged in MVPA (P < 0.001), and were more active both at school and in the home environment (Table 3). Although average counts/minute were the highest during recess as expected, overall, children spent relatively little recess time engaged in MVPA (12% in boys and 7% in girls).

**Table 3:** Physical activity during recess, school, and home time, and overall daily activity

Measurement period		Boys	Girls	P
		(n = 230)*	(n = 197)	
Recess	Time worn (mins/d)	74 (9)	75 (8)	0.848
	Average counts/min	1518 (419)	1108 (321)	<0.001
	MVPA (mins/d)	9 (6)	5 (4)	<0.001
School	Time worn (mins/d)	343 (45)	342 (44)	0.671
	Average counts/min	717 (156)	596 (145)	<0.001
	MVPA (mins/d)	17 (10)	11 (7)	<0.001
Home	Time worn (mins/d)	315 (50)	319 (51)	0.162
	Average counts/min	795 (302)	690 (248)	0.004
	MVPA (mins/d)	16 (11)	12 (9)	<0.001
Total	Time worn (mins/d)	741 (173)	728 (170)	0.412
	Average counts/min	713 (179)	614 (155)	<0.001
	MVPA (mins/d)	32 (17)	23 (14)	<0.001

All data are presented as mean (SD).

*n refers to number of children with 2-3 valid full days of data and 4-5 days of recess data.

The number of playground facilities in school was significantly associated with physical activity in children (Table 4). During recess, average counts per minute were 3.2% (95% CI 0.0-6.4%) higher with 8.3% (0.8-16.3%) more minutes of MVPA for every 10 additional playground facilities, once adjusted for age, sex, school roll, PA policies and weather. The corresponding differences during total school time did not reach statistical significance for either average counts (P = 0.072) or minutes of MVPA (P = 0.093). The amount of physical activity during the "home" hours was significantly related to the number of playground facilities within the school environment as well, with average counts being 5.6% (3.5-7.7%) and minutes of MVPA being 10.5% (5.5-15.7%) higher for every 10 additional facilities.

**Table 4:** The effect of play facilities on recess, school, and home time, and total physical activity

		Ratio	95% CI	P
Recess time	Average counts	1.032	1.000, 1.064	0.049
	MVPA	1.083	1.008, 1.163	0.033
School time	Average counts	1.023	0.998, 1.048	0.072
	MVPA	1.067	0.988, 1.153	0.093
Home time	Average counts	1.056	1.035, 1.077	<0.001
	MVPA	1.105	1.055, 1.157	<0.001
Total activity	Average counts	1.038	1.021, 1.056	<0.001
	MVPA	1.075	1.016, 1.138	0.015

Data are presented as ratios per 10-unit increase in play facilities adjusted for age, sex, school roll, school policies surrounding physical activity, and weather (daily rain and temperature).

Differences in school physical activity policies were not associated with physical activity, either in terms of average counts per minute or minutes of MVPA (Table 5), once adjusted for other factors in the model.

**Table 5:** The effect of school policies pertaining to physical activity on recess, school, and home time, and total physical activity

		Ratio	95% CI	P
Recess time	Average counts	1.002	0.990, 1.014	0.719
	MVPA	1.012	0.981, 1.044	0.433
School time	Average counts	1.004	0.995, 1.013	0.330
	MVPA	1.016	0.986, 1.047	0.278
Home time	Average counts	1.002	0.996, 1.008	0.521
	MVPA	1.005	0.990, 1.020	0.505
Total activity	Average counts	1.007	1.000, 1.014	0.048
	MVPA	1.005	0.984, 1.027	0.615

Data are presented as ratios per 1-unit increase in the school policy scale adjusted for age, sex, school roll, number of permanent play facilities, and weather (daily rain and temperature).

## Discussion

Our data demonstrate that the number of permanent play facilities in a school playground is related to physical activity in children, when assessed using an objective measure of physical activity. Each additional 10-unit increase in facilities was associated with 3.8% more activity overall (counts/minute) and 7.5% more MVPA over the course of a day and 3.2% (counts/minute) and 8.3% (MVPA) during recess only. By contrast, policies around physical activity in the wider school environment were not related to activity in children, once adjusted for confounding variables.

Few other studies have examined the potential of fixed play structures to influence physical activity in children. Sallis *et al *[25] determined that the presence of permanent improvements such as soccer goals, basketball hoops, tennis courts and baseball diamonds was related to school-based activity in children, particularly if high levels of supervision were also apparent. An observational study of a single playground demonstrated that children were more likely to be very active in the more equipped areas (particularly the girls) when compared to the open spaces [24]. Previous work from our group has demonstrated that each additional facility was associated with 3.4% more MVPA (9 minutes a day) [26], which has also been confirmed in a Danish sample (Nielsen, personal communication). Despite the cross-sectional nature of existing data, these studies consistently report small but significant effects of playground structures on physical activity in children. However, whether altering a school playground is a viable intervention initiative is of interest. Only one intervention appears to have altered the physical structure of school playgrounds to determine the potential effect on activity [23,30]. High deprivation schools in the UK received soccer goals and basketball hoops as well as funding to create a quiet area and fencing around a separate sports area. Intervention children participated in 4% more moderate and 2.4% more vigorous activity during recess at 6 months [30], although the intervention effect was no longer significant at 12 months [23].

The observation that the number of school playground facilities was also associated with home-time activity was surprising, although several explanations are possible. Firstly, "home" activity in the context of this paper refers simply to after school time rather than to children being off the school premises. Therefore "home" activity could include children playing at school after hours (common in New Zealand) or being involved in after-school sporting activities. Alternatively, children who were more active at school also tended to be more active in home time (r = 0.25, P < 0.001).

Regardless of the consistent nature of these findings, all of the studies report only small differences in actual physical activity. For example, in our study, a 7.5% difference in MVPA per 10 additional playground structures translated to an additional 2 minutes of MVPA each day. Similarly, the intervention of Ridgers *et al *resulted in 4% more MVPA during recess, or 3 more minutes of the total 78 minute period [23,30]. Levels of total MVPA per day were low in our participants (23 minutes in girls and 32 minutes in boys), but comparable with other studies [31] that have used the Puyau *et al *[29] cut-offs in this age group. Other studies clearly show that choice of cut-off can have a marked effect on the time spent in categories of activity intensity [32,33].

Our study is cross-sectional in design, with a relatively small sample size (n = 16 schools). This meant that adjustment for further confounders such as participation in organized sport or school provision of portable sports equipment was not possible. However, in our sample, children who participated in organized sport for at least 20 minutes each week were not more physically active than children not involved in sport. Similarly, the amount of portable sports equipment adjusted for school roll was not related to overall activity (P = 0.631) or levels of MVPA (P = 0.707). A limitation of our study may have been the choice of a 60-second epoch length, as it is commonly considered that shorter epoch lengths will provide a more accurate reflection of the intermittent nature of children's activity [34]. However, even though significant differences have been observed in MVPA with different epoch lengths, findings have not been consistent [35], and are considered to be small and of unknown biological significance [32]. A further limitation is that the relative effect of different types of play structures could not be evaluated. We had 23 different categories of play items, meaning that an extremely large sample would be required to elucidate whether any particular structures were more efficacious. A sample of only 16 schools may also be insufficient to examine the potential for school policies to affect physical activity, particularly given the low variability in school policy that we observed. By contrast, studies which have demonstrated that policy can affect reported physical activity or fitness tend to be in much larger samples [12-14].

Advantages of our study include the use of objective measures of physical activity assessment, over multiple days (5 days of recess or 3 complete days). While a longer period of monitoring may be advantageous to estimate usual activity in all children, three days of complete measurement is seen as adequate [36,37], particularly given that individual measurements were obtained in more than 400 children. Considerable variability was observed in the playground counts (n = 30 to 135) between schools, but the playground measure itself performed well, demonstrating an intra-class reliability of 0.89. Lastly, all measurements were adjusted for weather, which can affect participation in physical activity in children [38,39].

In conclusion, a greater number of permanent play facilities in school playgrounds is associated with higher levels of physical activity in children. Whether altering the school playground offers a viable and perhaps sustainable alternative for promoting physical activity at school remains to be determined.

## Competing interests

The authors declare that they have no competing interests.

## Authors' contributions

RWT was the principal investigator of the project, conceived the idea for the study, was involved in study design, monitored data collection, contributed to analysis and interpretation of data and drafted and revised the paper. VLF undertook the data collection, completed all accelerometry analyses, and revised the draft paper.

SLC undertook the data collection and revised the draft paper. KM-J contributed to the physical activity aspects of the study and revised the draft paper. SMW contributed to study design, completed all statistical analyses and revised the draft paper. JIM contributed to study design and revised the draft paper. All authors read and approved the final manuscript.
